# Dermoscopy of Lupus Miliaris Disseminatus Faciei: A Step Closer to Diagnosis

**DOI:** 10.5826/dpc.1003a55

**Published:** 2020-06-29

**Authors:** Payal Chauhan, Rashmi Jindal, Nadia Shirazi

**Affiliations:** 1 Department of Dermatology, Himalayan Institute of Medical Sciences, Dehradun, India; 2 Department of Pathology, Himalayan Institute of Medical Sciences, Dehradun, India

**Keywords:** lupus miliaris disseminatus faciei, dermoscopy, granulomatous disorders, doxycycline

## Introduction

Lupus miliaris disseminatus faciei (LMDF) is an uncommon chronic inflammatory and granulomatous dermatosis, the exact etiology of which is hitherto unknown. LMDF typically manifests as yellowish red to brown papules on the face, particularly around the eyelids [1]. We present a case of LMDF and describe the dermoscopic findings of the case with histological correlation.

## Case Presentation

**Figure 1:**
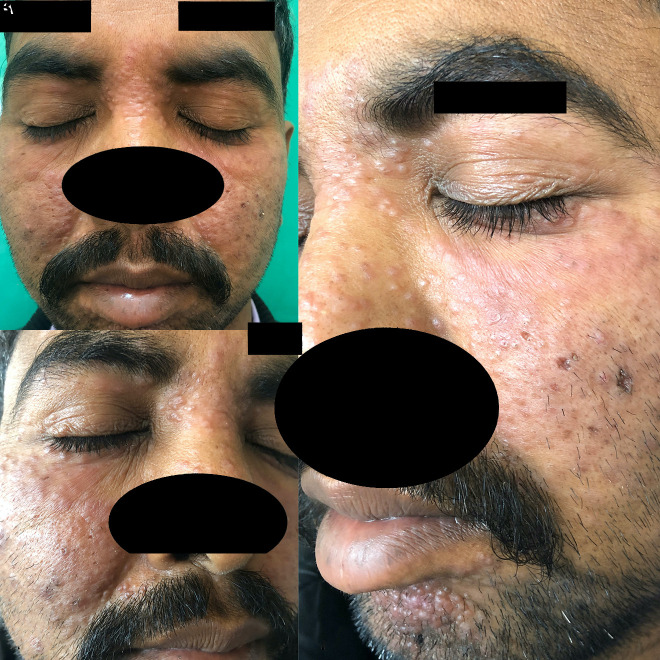
(A) Multiple, discrete reddish brown papules over both cheeks, nose, eyelids, and forehead; (B) closer view showing clustering of papules over upper and lower eyelids, perinasal areas with a few pitted scars over the right cheek; and (C) grouped reddish brown papules over the chin, periocular and perinasal areas with scars seen over the left cheek, a few of the papules coalescing to form plaques over the left cheek.

A 32-year-old-man presented to the dermatology department with complaints of multiple raised reddish lesions over the face for the preceding 8 months. There was no history of redness of the face, photosensitivity, or other skin lesions. On mucocutaneous examination, multiple, discrete, reddish brown papules and plaques were seen over both cheeks, forehead, nose, and chin. There was clustering of lesions around the chin and periocular, perinasal, and perioral areas. No surface change, background erythema, or telangiectasia was seen. A few pitted scars were also present on both cheeks (Figure 1, A–C).

**Figure 2:**
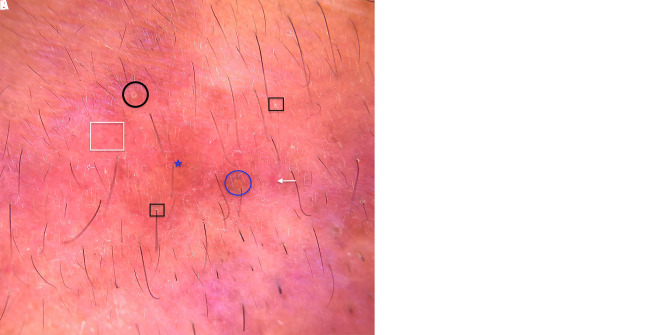
(A,B) Dermoscopic examination under polarized light (DermLite II Hybrid M, 3Gen, San Juan Capistrano, CA, USA; magnification ×10, attached to DermLite iPhone X adapter) of the papule revealed perifollicular structureless yellow to orangish brown areas (blue stars), with yellow (blue circles) or white (black circles) follicular keratotic plugs and perifollicular scales (black boxes). Unfocused arborizing (white arrows) and (B) linear vessels (white box) present over reddish-brown background.

Dermoscopic examination (DermLite II Hybrid M Dermatoscope, magnification ×10 in polarized mode) revealed multiple, ill-defined, yellow to orangish brown, structureless areas present perifollicularly, few of which were filled with yellow or white keratotic follicular plugs (Figure 2, A and B).

**Figure 3:**
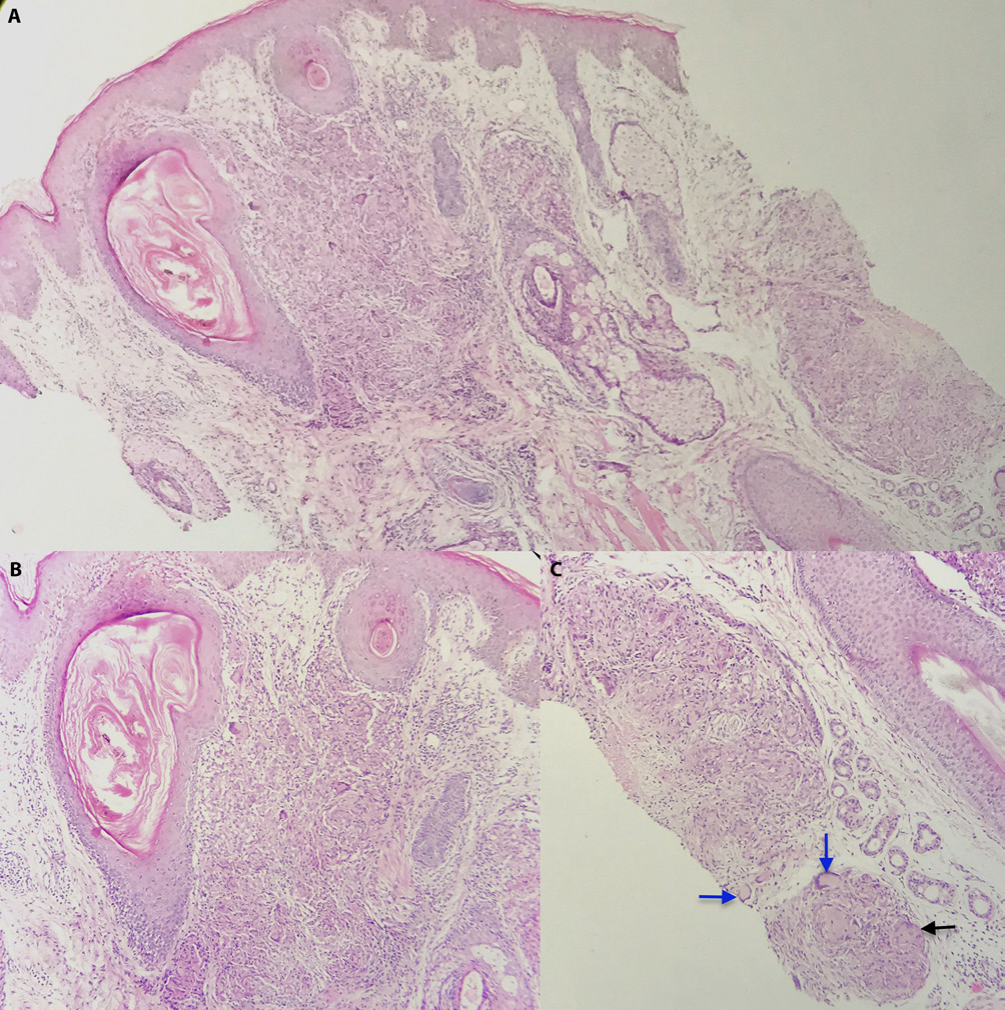
(A) Histopathological examination showing acanthosis and follicular plugging in epidermis. Dermis shows well-defined granulomas with perifollicular localization predominantly (H&E, ×10). (B) Perifollicular granulomas composed of predominantly epithelioid cells, a few lymphocytes, and giant cells in dermis (H&E, ×20). (C) Higher power showing Langhans (blue arrows) and foreign body giant cells (black arrow) with focal caseous necrosis (H&E, ×40).

Histopathological examination of the patient revealed acanthotic epidermis with follicular plugging. Dermis showed well-defined, predominantly perifollicular, epithelioid cell granulomas, focal caseous necrosis, and Langhans and foreign body giant cells (Figure 3, A–C).

Stains done for microorganisms (PAS, ZN) were negative. Routine laboratory investigations including Mantoux test, chest x-ray, and serum angiotensin-converting enzyme levels were all within normal limits. A diagnosis of LMDF was made after clinicopathological correlation and the patient was started on tablet doxycycline 100 mg twice a day for 6 weeks. Although the patient did not follow up in person, upon telephonic inquiry he reported significant improvement and doxycycline was tapered to 100 mg once a day for another 4 weeks.

## Conclusions

The dermoscopic findings of follicular keratotic plugs and vessels over brownish yellow areas seen in our case are similar to those previously reported [1]. Ayhan et al noted follicular keratotic plugs with vascular structures to be the predominant dermoscopic findings in cases described by them [1]. We believe that the reddish brown background seen in dermoscopy mirrors the dermal granulomas present in histology, with perifollicular structureless yellow to orangish brown areas representing the perifollicular localization of granulomas. Keratotic follicular plugs on dermoscopy are believed to be secondary to the follicular hyperkeratosis and lateral pressure on follicles by the surrounding granulomas [1]. Follicular plugs were seen under both the dermatoscope as well as microscope in the present case. LMDF needs to be differentiated from other disorders such as papular sarcoidosis, granulomatous rosacea, lupus vulgaris, and post kala-azar dermal leishmaniasis. Perifollicular localization of structureless yellow to orangish brown areas along with follicular keratotic plugs helps differentiate LMDF from its clinical mimickers such as sarcoidosis and lupus vulgaris, where—although yellowish orange areas are seen—they are not localized perifollicularly nor are keratotic plugs a prominent finding [1]. The absence of underlying vascular polygons would help in differentiating LMDF from granulomatous rosacea where vascular polygons are characteristically seen. Treatment of LMDF poses a challenge to physicians and is often unsatisfactory. The disease runs a protracted course, healing with unsightly scars over several months to years (12–24 months usually). Early treatment is warranted to prevent resolution of the disease with depressed scars [2]. Although tetracyclines are considered first-line agents in the management of LMDF, response can be variable [2,3]. Other treatment modalities such as isotretinoin, dapsone, corticosteroids, and clofazimine have also been reported to be effective in some patients [3]. In the present case, doxycycline was preferred over minocycline because the former is more cost-effective.

We present this case to describe dermoscopic findings in LMDF and their histological correlation. On a detailed literature search, we came across only one previous report focusing on the dermoscopic aspect of LMDF. Dermoscopy can act as a valuable, real-time, bedside, auxiliary tool aiding the physician in reaching the diagnosis of this uncommon disorder. Dermoscopy of differential diagnosis of LMDF is summarized in Table 1.

## Figures and Tables

**Table 1:** Dermoscopic Findings of Differential Diagnosis of Lupus Miliaris Disseminatus Faciei

Disorder	Dermoscopic Findings
Sarcoidosis	Orangish areas with well-focused linear and branching vessels; white scar-like depigmented areas
Granulomatous rosacea	Linear reddish or purple vessels arranged in polygonal network (vascular polygons) with diffuse or localized orangish areas
Lupus vulgaris	Focal or diffuse orange areas with focused linear or branching vessels; milia-like cysts, pigmentation structures, whitish reticular streaks less commonly seen
Post kala-azar dermal leishmaniasis	Multiple yellow tears and erythema
Hansen disease	Diminished pigment network with yellowish orange areas in borderline tuberculoid spectrum; whitish yellow structureless areas with vessels in histoid leprosy; blanched erythema, follicular plugging, yellow-orange areas in type 1 reaction; increased erythema with dilated vessels in type 2 lepra reaction
Present case	Ill-defined, structureless, perifollicular, yellow to orangish brown areas with white or yellow keratotic follicular plugs; few linear and arborizing vessels over reddish brown background
